# Comparison and development of machine learning tools in the prediction of chronic kidney disease progression

**DOI:** 10.1186/s12967-019-1860-0

**Published:** 2019-04-11

**Authors:** Jing Xiao, Ruifeng Ding, Xiulin Xu, Haochen Guan, Xinhui Feng, Tao Sun, Sibo Zhu, Zhibin Ye

**Affiliations:** 1 Department of Nephrology, Huadong Hospital Affiliated To Fudan University, Shanghai, 200040 China; 2 Shanghai Key Laboratory of Clinical Geriatric Medicine, Huadong Hospital Affiliated To Fudan University, Shanghai, 200040 China; 3 School of Medical Instrument and Food Engineering, University of Shanghai for Science and Technology, Shanghai, 200093 China; 4 MOE Key Laboratory of Contemporary Anthropology, School of Life Sciences, Fudan University, Shanghai, 200438 China

## Abstract

**Background:**

Urinary protein quantification is critical for assessing the severity of chronic kidney disease (CKD). However, the current procedure for determining the severity of CKD is completed through evaluating 24-h urinary protein, which is inconvenient during follow-up.

**Objective:**

To quickly predict the severity of CKD using more easily available demographic and blood biochemical features during follow-up, we developed and compared several predictive models using statistical, machine learning and neural network approaches.

**Methods:**

The clinical and blood biochemical results from 551 patients with proteinuria were collected. Thirteen blood-derived tests and 5 demographic features were used as non-urinary clinical variables to predict the 24-h urinary protein outcome response. Nine predictive models were established and compared, including logistic regression, Elastic Net, lasso regression, ridge regression, support vector machine, random forest, XGBoost, neural network and k-nearest neighbor. The AU-ROC, sensitivity (recall), specificity, accuracy, log-loss and precision of each of the models were evaluated. The effect sizes of each variable were analysed and ranked.

**Results:**

The linear models including Elastic Net, lasso regression, ridge regression and logistic regression showed the highest overall predictive power, with an average AUC and a precision above 0.87 and 0.8, respectively. Logistic regression ranked first, reaching an AUC of 0.873, with a sensitivity and specificity of 0.83 and 0.82, respectively. The model with the highest sensitivity was Elastic Net (0.85), while XGBoost showed the highest specificity (0.83). In the effect size analyses, we identified that ALB, Scr, TG, LDL and EGFR had important impacts on the predictability of the models, while other predictors such as CRP, HDL and SNA were less important.

**Conclusions:**

Blood-derived tests could be applied as non-urinary predictors during outpatient follow-up. Features in routine blood tests, including ALB, Scr, TG, LDL and EGFR levels, showed predictive ability for CKD severity. The developed online tool can facilitate the prediction of proteinuria progress during follow-up in clinical practice.

**Electronic supplementary material:**

The online version of this article (10.1186/s12967-019-1860-0) contains supplementary material, which is available to authorized users.

## Background

Chronic kidney disease (CKD) is associated with an increased risk for adverse clinical events, which makes it a major public health problem worldwide [1]. Although it is well recognized that CKD is independently associated with increased risks for end stage renal disease, cardiovascular events, and all-cause mortality, the prognosis for individual patients still lacks sufficient information [2]. Clinically usable strategies for the risk stratification of each outcome are important for making treatment decisions [3, 4].

Renal prognosis predictive models in CKD patients may be helpful in identifying those at high risk who may benefit from more intensive management, such as higher doses of RAAS (renin–angiotensin–aldosterone system) inhibitors, anticoagulation therapy, and intensive blood glucose, blood pressure, urate and lipid-lowering medications [5]. In addition, how to screen outpatient CKD patients who should have intensive and quick examinations is of great clinical and economical significance. With the use of such models, most patients with risks of having proteinuria less than 1 g/24 h can be stratified as low risk and can potentially be treated solely by their primary outpatient follow-up, whereas those at high risk (proteinuria more than 1 g/24 h) can be referred to urgent care by an inpatient management registration. Similarly, models predicting renal progression may identify patients at low risk for renal failure in the next 5 years, for whom advanced treatment may be inappropriate [6]. Proteinuria has always been recognized as the most important risk factor [7]. A recent study improved the prediction efficacy by using proteinuria to estimate the glomerular filtration rate [8]. However, models using proteinuria need to collect the 24-h urine, which is inconvenient, especially in outpatient clinics.

Studies have been conducted to try to use routinely obtained laboratory tests without proteinuria to predict renal progression. Models including age, sex, estimated GFR, albuminuria, serum calcium, serum phosphate, serum bicarbonate, and serum albumin can accurately predict the progression to kidney failure in patients with CKD stages 3–5 [4]. More recently, artificial intelligence approaches have been proven to solve real problems, including rule-based and gold standard oriented diagnoses or prognoses. To help clinicians select prediction tools for predicting the severity of CKD, we established and compared nine prediction models using statistical, machine learning and neural network approaches with blood-derived outpatient clinical features and demographic features. Based on the results, we further established an online tool for patient follow-up urinary protein severity prediction.

## Methods and materials

### Patients and data pre-processing

A total of 551 pathologically confirmed CKD patients with 24-h urinary protein were recruited from August 2015 to September 2018 at the Department of Nephrology in the Shanghai Huadong Hospital Affiliated to Fudan University. None of the patients were diagnosed with METS, cancers or cardio- and cerebrovascular diseases. The detailed demographic characteristics of the cohort are listed in Table 1. In this study, urine protein > 1 g/24 h was used as the outcome variable to classify the progress and severity of proteinuria in patients with kidney disease. Our study was approved by the Clinical Ethics Review Committee of the Shanghai Huadong Hospital Affiliated to Fudan University, and clinical consent was obtained from all patients. We first cleaned and formatted the data before model fitting. Then, in the pre-processing stage, we transformed categorical variables into binary dummy variables. Finally, we scaled the data as most models are affected by the difference in the scale of the variables. We performed power analysis over urinary protein values to determine if the sample size was suitable for further statistical process (alpha = 0.05). All values were normalized to reduce the dimension-introduced bias using Z-score standardization as previously described [9–13]: (Eq. 1).

1 $$ z = \frac{x - \mu }{\sigma } $$

where μ is the average of the features across all samples, and α is the standard deviation.

**Table 1:** Demographic data of 551 patients

	Cases (n = 551)			
	No	Percent	Mean	SD
Age (years)			58.15	16.45
≤ 58.15	251	45.54		
> 58.15	300	54.46		
Range	18–90			
Sex				
Male	283	51.3		
Female	268	48.7		
Height (cm)			165.67	8.28
≤ 165.67	279	50.64		
> 165.67	272	49.36		
Range	145–190			
Weight (g)			67.09	12.84
≤ 67.09	286	51.91		
> 67.09	265	48.09		
Range	39–118			
BMI			24.33	3.67
≤ 24.33	298	54.08		
> 24.33	253	45.92		
Range	16.23–41.32			
CRP (mg/L)			7.32	15.01
≤ 7.32	379	68.78		
> 7.32	147	26.68		
Missing	25	4.54		
Range	0–190			
ALB (g/L)			37.72	6.65
≤ 37.72	222	40.29		
> 37.72	328	59.53		
Missing	1	0.18		
Range	13–66			
TC (mmol/L)			4.88	1.54
≤ 4.88	310	56.26		
> 4.88	231	41.92		
Missing	10	1.81		
Range	1.30–12.93			
TG (mmol/L)			1.91	1.46
≤ 1.91	348	63.16		
> 1.91	193	35.03		
Missing	10	1.81		
Range	0.4–18.2			
BG (mmol/L)			5.13	1.59
≤ 5.13	377	68.42		
> 5.13	173	31.40		
Missing	1	0.18		
Range	2.3–17.5			
BUN (mmol/L)			10.42	8.35
≤ 10.42	393	71.32		
> 10.42	157	28.49		
Missing	1	0.18		
Range	2.5–62.0			
EGFR (ml/min)			57.95	35.63
≤ 57.95	275	49.91		
> 57.95	276	50.09		
Range	1.0–154.4			
Scr (umol/L)			192.57	212.21
≤ 192.57	410	74.5		
> 192.57	141	25.5		
Range	41.9–1460.7			
SUA (umol/L)			394.41	110.63
≤ 394.41	285	51.72		
> 394.41	266	48.28		
Range	49.0–808.0			
SK (mmol/L)			4.03	0.47
≤ 4.03	306	55.54		
> 4.03	241	43.74		
Missing	4	0.72		
Range	2.7–5.6			
Sna (mmol/L)			142.13	3.08
≤ 142.13	289	52.45		
> 142.13	258	46.82		
Missing	4	0.72		
Range	108.2–152.0			
LDL (mmol/L)			2.80	1.12
≤ 2.80	325	58.98		
> 2.80	226	41.02		
Range	0.42–8.95			
HDL (mmol/L)			1.30	0.37
≤ 1.30	317	57.53		
> 1.30	233	42.29		
Missing	1	0.18		
Range	0.53–2.81			
Uprotein (g/24 h)			1.55	2.21
≤ 1.0	330	59.89		
> 1.0	221	40.11		
Range	0–20.8			

### Establishment of a predictive model

In this study, nine predictive models were established to predict the progression of urinary protein in patients with chronic kidney disease, and model selection was based on several currently and frequently adopted predictive model types. For the linear model, the logistic regression model (LR) [14, 15], the elastic network model (Elastic Net) [16–18], the lasso regression model (Lasso) [19], and the ridge regression model (Ridge) were selected [20–22]. The neural network model (NN) [23] was chosen because it is an important class of nonlinear prediction models [24] and has been reported to predict CKD. For the kernel-based model, a support vector machine (SVM) with a Gaussian kernel (RBF) has been widely adopted in many clinical applications, such as coronary artery disease prediction [25, 26]. For the decision tree approach, the random forest (RF) model [27–29] and the XGBoost model [30–32] have also been used in clinical research. Finally, a basic prediction technique [33], k-nearest neighbor algorithm (k-NN) was built [34]. The model was fitted using the method described above for each set of parameters, which were adjusted to obtain the average performance index. The log-loss was calculated to indicate the confidence of the model. The lower the log-loss value is, the more confident the model is for its classification results [35, 36]. The technical parameters of the selected prediction models are listed for the optimization of the equations (Table 2). Model establishment and brief illustrations are described in Additional file 1.

**Table 2:** Tuning parameters of the predictive models

Models	Tuning
LR	α (Regularization parameter)
Elastic Net	γ (Mixing percentage), α (Regularization parameter)
Lasso	α (Regularization parameter)
Ridge	α (Regularization parameter)
SVM	γ (Gaussian kernel), C (Cost)
RF	n_estimators (#subtrees)
k-NN	k (#Neighbors)
NN	Size (#hidden layer units), α (Regularization parameter)
XGBoost	Depth (maximum depth of number), weight (the smallest sample weight and weight in the child node)

### Assessment of models in CKD severity prediction

**Fig. 1:**
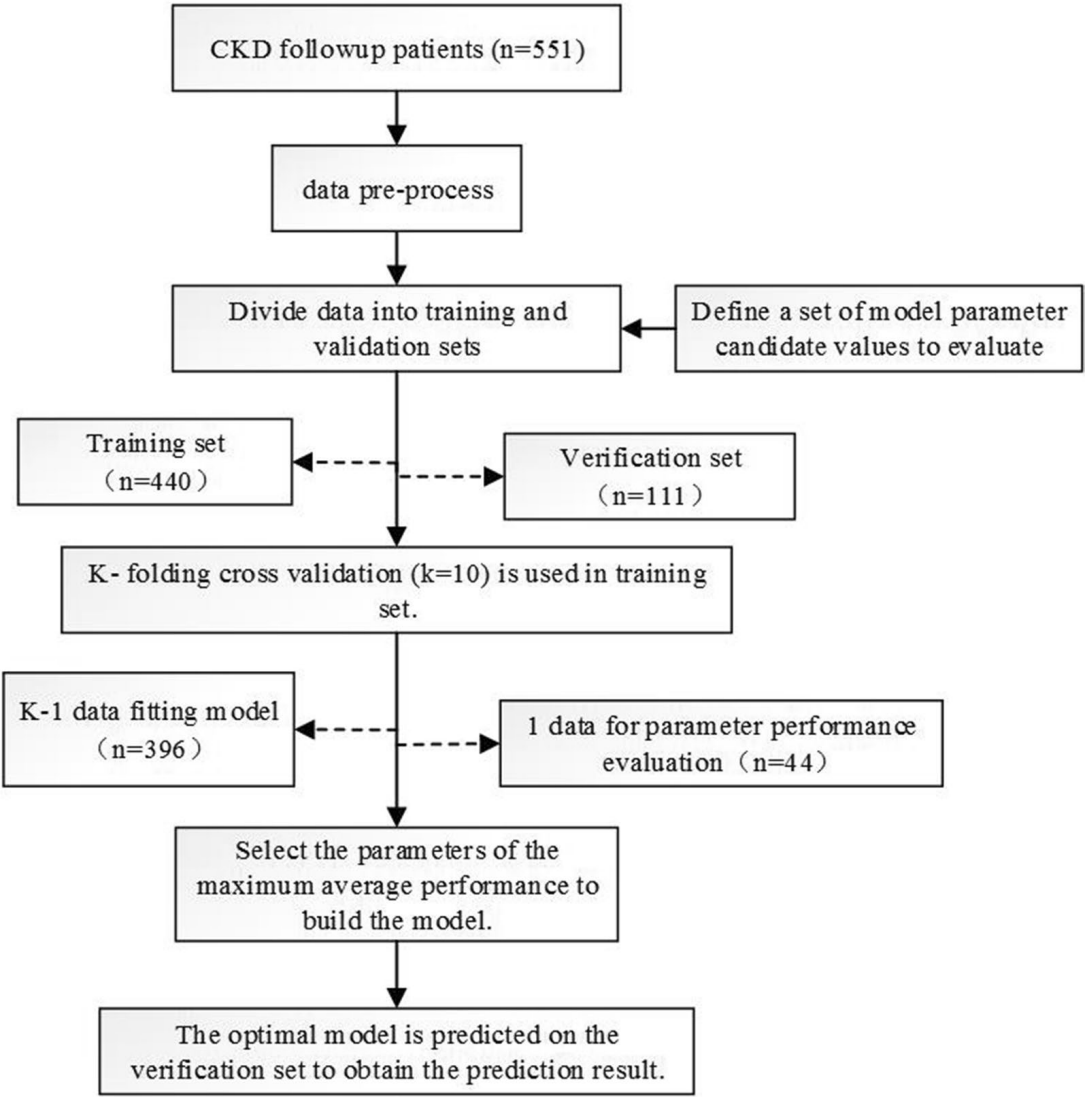
Model training, parameter adjustment and performance evaluation. 551 patients were recruited in the current study. The data were pre-processed and randomly divided into a training set (80%) and a validation set (20%), and the proportion of the two class proportions in each set is the same. In the training set, k-fold cross-validation (k = 10) is used, and various parameter combinations are exhausted by grid search. Performance evaluation indices such as AUC and AP were adopted to judge the average predictive performance of the model. The average performance maximum is used as the best performance tuning parameter, and the prediction is finally performed on the test set

In this study, we have improved the method of the data resampling technique [37] considering the overfitting problem caused by the empirical risk minimization algorithm of the optimization model. First, the candidate values of the model parameters were defined, and the patients were randomly allocated into a training set (80%) and a validation set (20%), where the two class proportions in each set were the same. In the training set, k-fold cross-validation (k = 10) was used, and various parameter combinations were exhausted by grid search. For each set of parameters, 9/10 of data were used for fitting the model in turn, and 1/10 of data was used for validation. AU-ROC was selected as the performance index, which was calculated 10 times, and its average performance was calculated as the parameter score of the current parameter combination. The average value of the parameter value grid was selected as the best performance adjustment parameter of the current iteration and was finally executed on the test set. A forecast flow chart is shown in Fig. 1. This step was repeated 20 times randomly, i.e., 20 resampling iterations were defined. This study used the same resampling to evaluate different models. For each model, the evaluation indicators used were the confusion matrix, area under the curve (AUC), sensitivity (recall), specificity, accuracy, log-loss, AP, F1, false positive rate (FPR), and precision. Each evaluation used the same data segmentation and repetition to ensure a fair comparison of the models. Additionally, we carried out hierarchical clustering analysis over methods based on false positive (FP) and false negative (FN) values. In this study, Python (version 3.7.0) and R (version 3.5.1) were used to build and evaluate the models.

To better evaluate the performance of the models, we further compared the AU-ROC from each resampling calculation using a paired *t* test. P < 0.05 was regarded as significant. In addition to performance comparisons, this study also analysed the importance of variable factors in the predictive models. For each model, the relative effect size was quantified by assigning a weight between 0 and 1 for each variable. The models XGBoost and RF allowed the importance of variables to be derived during model training; the coefficients of the Elastic Net, Lasso, and Ridge models were used as the importance factor. For models, such as kNN and SVM, wherein the importance of variables was difficult or impossible to extract, the mean decrease accuracy was obtained by directly measuring the effect of each feature on the accuracy of the model. Briefly, the model was fitted, and parameter adjustment was performed to predict the validation set to obtain the model performances. Then, the feature values were disturbed to establish a new disturbance prediction set. Obviously, for the unimportant variables, the scrambling order has little effect on the accuracy of the model, but for the important variables, the scrambled order will reduce the accuracy of the model. Finally, the relative importance ratio of all the eigenvalues was given a weight between 0 and 1 according to the overall proportion, thereby obtaining the effect sizes.

### Establishment of web tools for CKD severity prediction

To facilitate the predictive function in clinical practices, we designed and developed a CKD Prediction System for the above models whose predictive precision, sensitivity and specificity were highest. The proteinuria predictor was embedded in the web tool. User data interaction and visualization of analysis results were displayed using HTML5, JavaScript, and PHP. Source codes for model establishment by Python and web tools by PHP are provided in Additional file 1.

## Results

### Patients and variables

This study recruited 551 patients with CKD from the Department of Nephrology, Huadong Hospital, Shanghai Fudan University Affiliated Hospital who had pathologically confirmed 24-h urine protein. The training dataset included 330 mild CKD patients (urinary protein ≤ 1 g/24 h) and 221 moderate/severe CKD patients (urinary protein ≥ 1 g/24 h). Through statistical power analysis of the urinary protein values, the sample size in our study was competent for further procedures with power at 1. The following non-urine indicators of 13 outpatient blood biochemistry tests and 5 demographic features were used as predictive variables: CRP, ALB, TC, TG, BG, BUN, EGFR, Scr, SUA, SK, Sna, LDL, HDL, sex, age, height, weight, and BMI. Urine protein (g/24 h) was considered an outcome variable to judge the status of CKD patients.

### Tuning of parameters

**Fig. 2:**
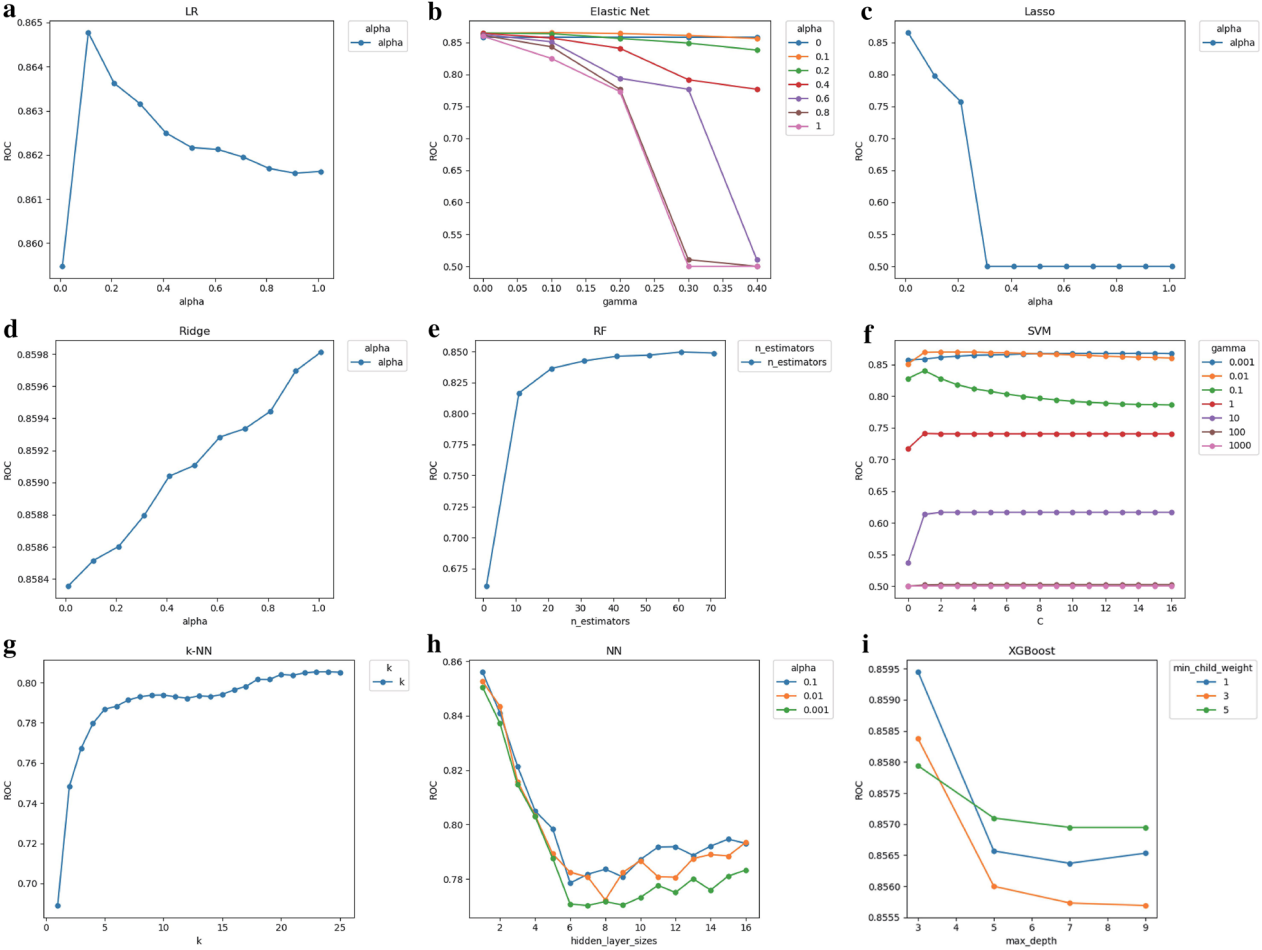
Tuning results of model parameters using re-sampling approach. **a**–**i** Five models have one adjustment parameter (LR, RF, Lasso, Ridge, and k-NN), and four models have two adjustment parameters (Elastic Net, SVM, NN and XGBoost). For each set of parameters, the model parameters were evaluated for fit using the procedure described in Flowchart 1. The optimal parameters for each model are selected by obtaining the parameters that the model evaluates to the maximum

The average AU-ROC for different models and their parameters are listed (Fig. 2). The SVM was not sensitive to cost choice C, and the kernel smoothing parameter σ of 0.01 was optimal. For k-NN, a relatively large number of k = 24 was optimal; for RF, a relatively large number of randomly selected 61 subtrees provided the best performance. The maximum depth (max_depth) of the XGBoost tree was 3, and the minimum leaf node sample weight (min_child_weight) of 1 achieved optimal performance.

### Validation of the training set

**Fig. 3:**
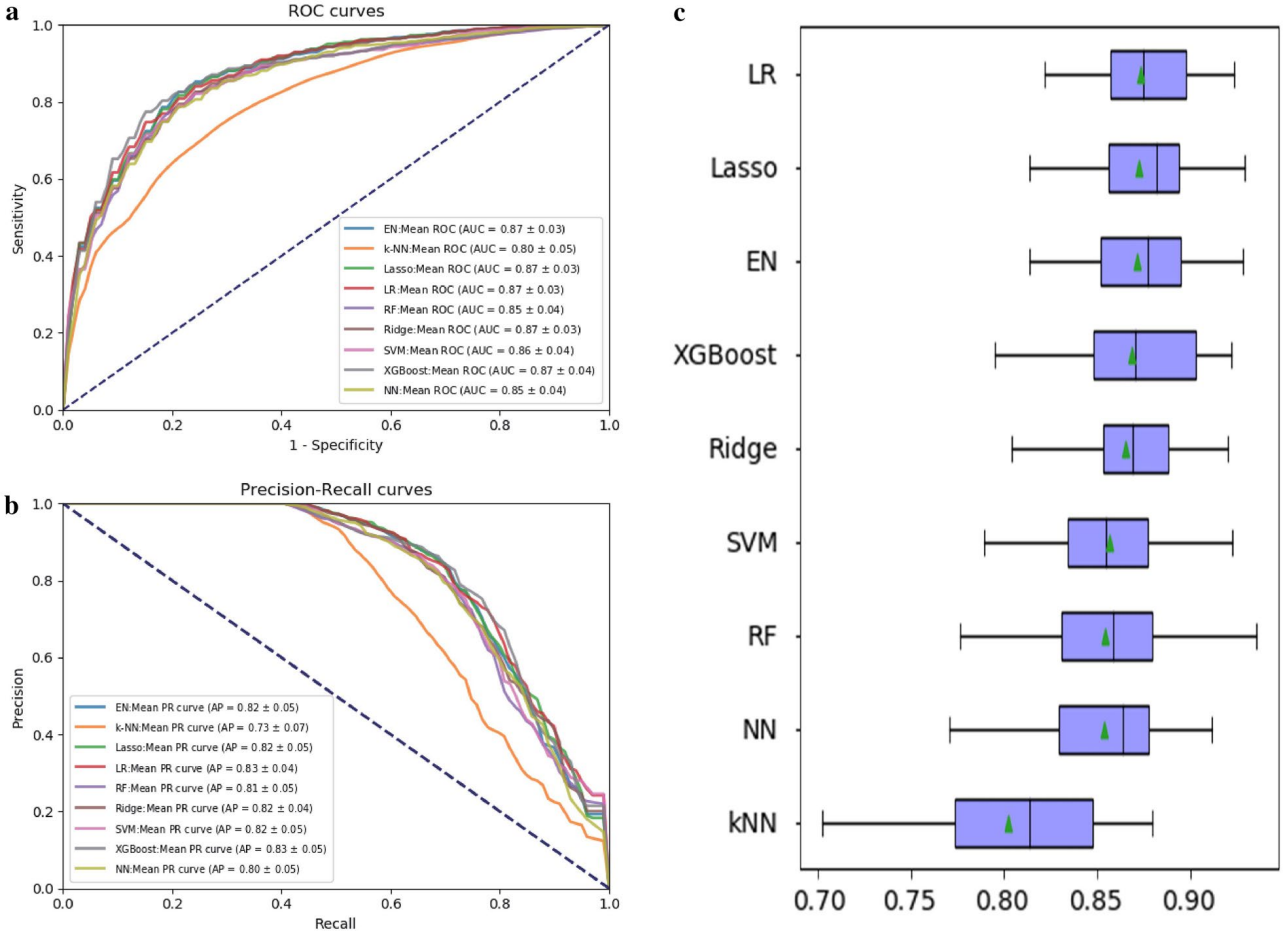
Evaluation of the predictive models. **a** The left picture showed the average ROC curves from of nine models in the validation sets. Mean AUC values with standard deviations of different prediction models were shown in the box. **b** The right picture showed average PR curves, indicating the tradeoff between precision and recall. Mean AP values with standard deviations of different prediction models were shown in the box. **c** The box plot is ranked according to the performance of the nine models using AU-ROC mean. The green triangle in the box stands for the mean values

The average ROC curves and PR curves during the 20-fold data resampling process are shown in Fig. 3a, b. Most models had AUC values above 0.85, but the value of k-NN was lower (0.80). We used the AP value as the criterion for the PR curve [38]. The APs of the Elastic Net, Lasso, LR, Ridge, SVM and XGBoost models were all above 0.82. The confusion matrix (rounding) was also calculated for the nine models (Table 3). As shown in Table 3, k-NN generated a large amount of FNs (= 12) and FPs (= 17) during the prediction process, while the other models had the same number of FNs, which could be controlled within 10, where the Lasso and Elastic Net models produced the least amount of FNs (= 7). The model XGBoost produced the minimum number of FPs (= 11).

**Table 3:** Confusion matrices of 9 models

Confusion matrix	Actual	Prediction	
		Uprotein ≤ 1.0 mg/24 h	Uprotein > 1.0 mg/24 h
LR	Uprotein ≤ 1.0 mg/24 h	37	8
	Uprotein > 1.0 mg/24 h	12	54
Elastic Net	Uprotein ≤ 1.0 mg/24 h	38	7
	Uprotein > 1.0 mg/24 h	14	52
Lasso	Uprotein ≤ 1.0 mg/24 h	38	7
	Uprotein > 1.0 mg/24 h	14	52
Ridge	Uprotein ≤ 1.0 mg/24 h	37	8
	Uprotein > 1.0 mg/24 h	14	51
SVM	Uprotein ≤ 1.0 mg/24 h	37	8
	Uprotein > 1.0 mg/24 h	13	53
RF	Uprotein ≤ 1.0 mg/24 h	37	8
	Uprotein > 1.0 mg/24 h	14	52
k-NN	Uprotein ≤ 1.0 mg/24 h	33	12
	Uprotein > 1.0 mg/24 h	17	49
NN	Uprotein ≤ 1.0 mg/24 h	37	8
	Uprotein > 1.0 mg/24 h	14	52
XGBoost	Uprotein ≤ 1.0 mg/24 h	37	8
	Uprotein > 1.0 mg/24 h	11	55

The nine methods were clustered based on hierarchical clustering analysis using the FP and FN values from one random sampling (Additional file 1: Figure S1). Similar models drew similar results; for example, the decision tree models XGBoost and random forest were clustered closely. Table 4 shows the AUC, sensitivity (recall), specificity, accuracy, log-loss, FP rate, precision, f1, and AP of each model evaluation result.

**Table 4:** Performance summary in terms of AU-ROC sensitivity (recall), specificity, accuracy, log-loss, FP rate, precision

Models	AUC	95%CI		Sensitivity (recall)	Specificity	Accuracy	log-loss	FP rate	Precision	AP	F1
		Lower bound	Upper bound								
LR	0.873	0.808	0.939	0.83	0.82	0.82	6.16	0.18	0.76	0.83	0.79
Elastic Net	0.871	0.805	0.937	0.85	0.80	0.82	6.29	0.20	0.74	0.82	0.79
Lasso	0.872	0.807	0.938	0.84	0.79	0.81	6.41	0.21	0.74	0.82	0.79
Ridge	0.865	0.798	0.933	0.83	0.79	0.81	6.71	0.21	0.73	0.82	0.78
SVM	0.857	0.786	0.928	0.82	0.81	0.81	6.50	0.19	0.75	0.82	0.78
RF	0.854	0.782	0.926	0.83	0.79	0.80	6.77	0.21	0.73	0.81	0.77
k-NN	0.802	0.721	0.883	0.74	0.74	0.74	8.91	0.26	0.69	0.73	0.70
NN	0.854	0.783	0.925	0.83	0.78	0.80	6.91	0.22	0.73	0.80	0.77
XGBoost	0.868	0.799	0.938	0.83	0.83	0.83	5.87	0.17	0.77	0.83	0.80

There were significant performance differences between the different models (Fig. 3c and Table 4). The linear models LR, Elastic Net, Lasso and Ridge had excellent performance, and the accuracy rate was up to 0.80. Among them, LR obtained the highest AUC value of 0.873, and the tree model XGBoost had an AU-ROC value of 0.868 and an accuracy rate of 0.83. K-NN obtained the lowest AUC value of 0.802. The best performance of sensitivity was the Elastic Net model, which is suitable for the early diagnosis of proteinuria progression in patients with chronic kidney disease. The best particularity was XGBoost and LR, which are suitable for the early stage of proteinuria in patients with chronic kidney disease. The sensitivity and specificity of the LR, Elastic Net, SVM, XGBoost and Lasso models both reached over 0.80. The XGBoost model had the lowest log-loss value (5.87), indicating that Lasso is more useful for its classification results, while the k-NN model had the highest log-loss value of 8.91. LR and XGBoost performed best regarding FP rate and precision, while XGBoost showed the highest AP values.

We further compared each model using the AU-ROC mean and paired t-test. Compared to the other models, LR, Elastic Net, Lasso, and XGBoost showed no statistical significance, implying that these models were similar in terms of their predictive power. In our study, k-NN provided the lowest predictive performance (Table 5).

**Table 5:** Comparison of AUCs. The upper part of the matrix represents the average AUC differences between models. The lower part represents statistical significance (p values) of paired t-tests

pVal\fold change	LR	Elastic Net	Lasso	Ridge	SVM	RF	k-NN	NN	XGBoost
LR	–	0.002	0.001	0.008	0.017	0.019	0.071	0.019	0.005
Elastic Net	0.195	–	− 0.001	0.006	0.015	0.017	0.069	0.017	0.003
Lasso	0.489	0.372	–	0.007	0.016	0.018	0.070	0.019	0.004
Ridge	0.001	0.014	0.001	–	0.009	0.011	0.063	0.011	− 0.003
SVM	0.000	0.001	0.000	0.055	–	0.002	0.054	0.003	− 0.012
RF	0.000	0.001	0.000	0.019	0.536	–	0.052	0.000	− 0.014
k-NN	0.000	0.000	0.000	0.000	0.000	0.000	–	− 0.052	0.066
NN	0.000	0.004	0.002	0.046	0.540	0.956	0.000	–	− 0.015
XGBoost	0.219	0.488	0.316	0.488	0.010	0.000	0.000	0.015	–

**Fig. 4:**
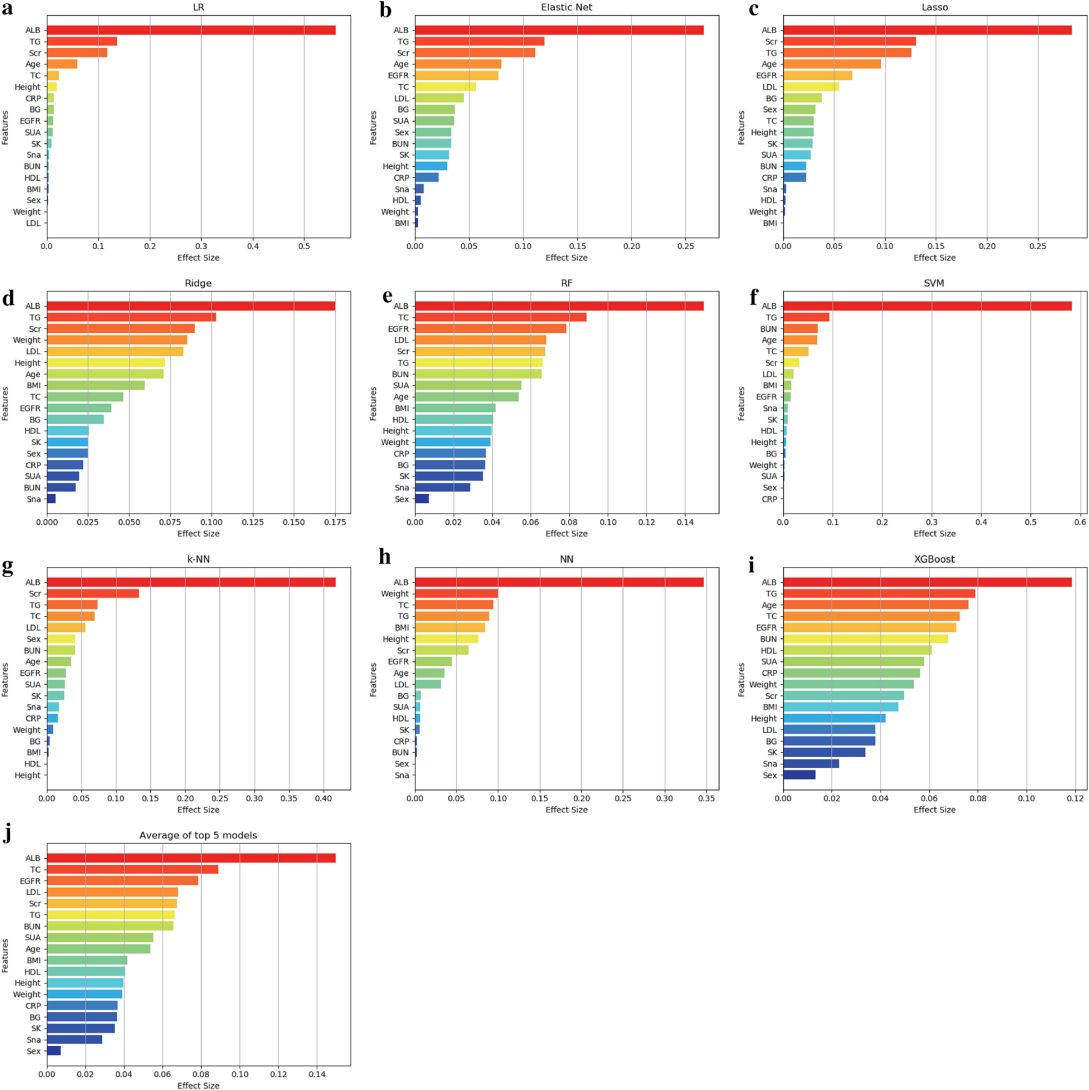
Factors effect size. The **a**–**i** histogram describes the proportion of factoric importance of different predictors in the model. For each model, the relative importance is quantified by assigning a weight between 0 and 1 for each variable. The models XGBoost and RF allow the importance of variables to be derived during model training; the coefficients of the Elastic Net, Lasso, and Ridge models are used as the basis for factor importance; the k-NN, LR, NN, and SVM models are obtained by the Mean decrease accuracy method. **j** The average factor importance of the top 5 models according to AU-ROC

The importance features, as shown in the effect sizes, were calculated (Fig. 4). For most of the models, the importance could be divided into two groups. The first group included ALB, Scr, TG, LDL, age, EGFR, and TC, which had important influences on the predictability of the models. The second group included BMI, height, weight and CRP, which showed less impact on prediction. ALB and TG were shown with the highest frequencies in the top predictors in all nine models, while Scr, TC, age and LDL were also shown with a high effect size in more than half of the models.

### Establishment of the website

**Fig. 5:**
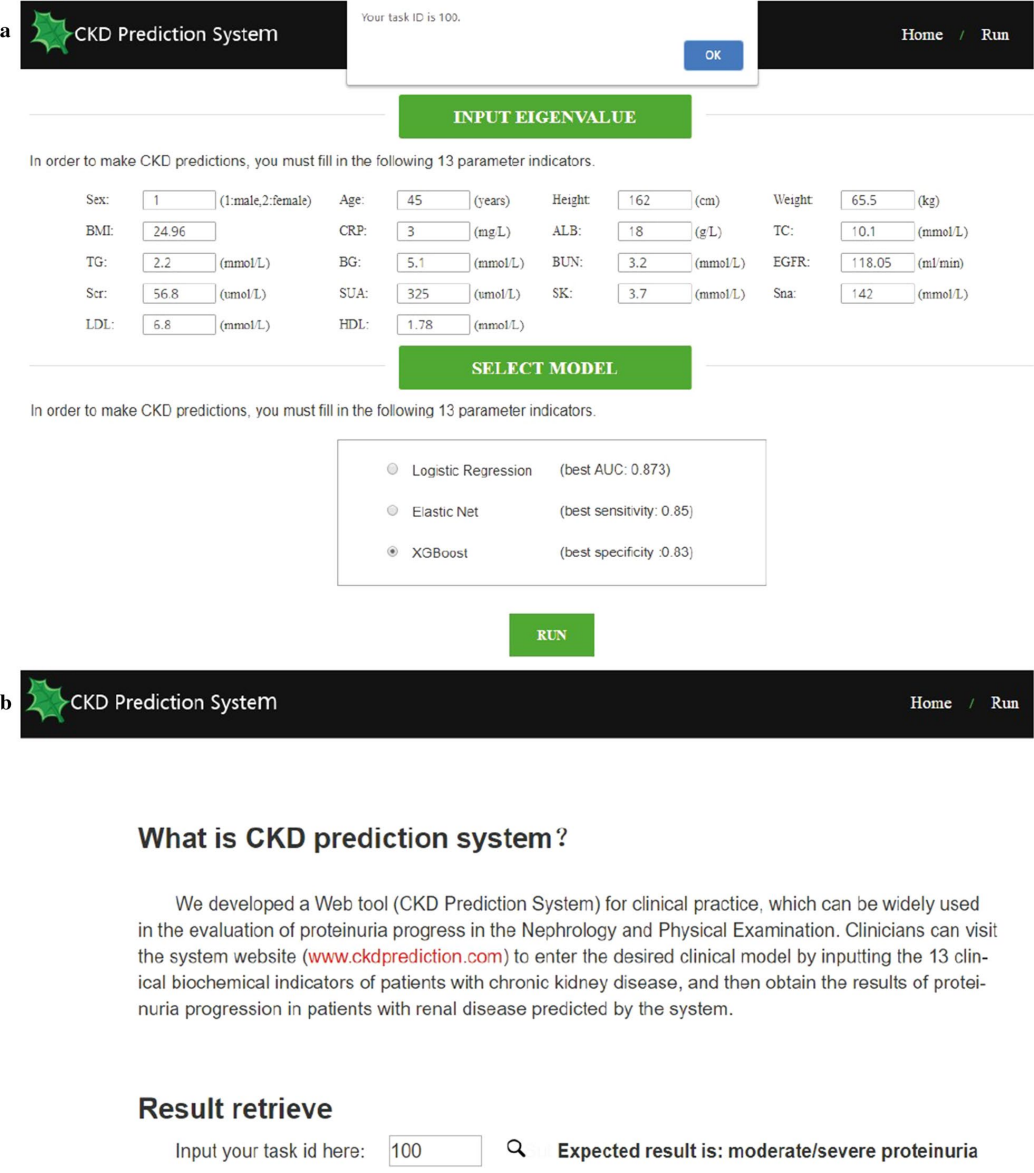
**a**, **b** Website-CKD Prediction System. 18 clinical features from patients can be input as values to predict the severity of CKD

In this study, we developed a Web tool (CKD Prediction System) for clinical practice that can be widely used in the evaluation of proteinuria progress in nephrology and during follow-up examinations (Fig. 5). Clinicians can visit the system website (http://www.ckdprediction.com) and use the desired clinical model by entering the 13 clinical biochemical indicators and 5 demographic features from follow-up CKD patients. The calculated probability of CKD progression will be predicted and obtained by the system. For example, after we input the features into the CKD Prediction System, the tool will feed back the prediction of the patient’s current status with “mild” or “moderate/severe”.

## Discussion

In this study, we applied 13 blood and 5 demographic parameters to predict the progression status of CKD by the severity of proteinuria using nine models. The linear models LR, Elastic Net, Lasso, Ridge and XGBoost met clinical needs and provided rapid screening for outpatients. Renal progression prediction is important in clinical practice for screening patients who are at a higher risk for renal failure. Various models have been developed and evaluated. Most models rely on the extent of proteinuria [39, 40]. However, measurement of 24-h proteinuria is not very applicable in real outpatient practice. Some assessed the changes in dipstick proteinuria, suggesting that changes in proteinuria over 2 years may be appropriate for the risk prediction of ESRD (end-stage renal disease) [41]. However, this model requires re-examination data from the patients, which could not be predicted at the first time of the patient’s visit.

Asif Salekin and John Stankovic [24] introduced the method of detecting CKD by using k-NN, RF and NN, analysed the characteristics of 24 clinical indicators, and sorted their predictability. Five indicators were identified for model construction, and a new CKD detection method (with or without CKD) was identified. Lin Lijuan et al. [42] analysed the risk factors of CKD progression in three stages of chronic kidney disease. The multi-factor analysis method in SPSS was used to study the effect of blood pressure control on the progression of CKD elderly patients. Patients with kidney disease have mutual influence, and the increased risk of CKD kidney injury in the elderly is related to the level of systolic blood pressure.

Unlike many studies using models to judge CKD from normal subjects, we hereby use machine learning and data mining to predict the patient’s CKD status. Similarly, Chase et al. [43] used six laboratory values (haemoglobin, bicarbonate, calcium, phosphorous, and albumin) in addition to EGFR to predict the probability of CKD patients progressing from phase 3 to phase 4 using naive Bayes and logistic regression. However, the sensitivity of the established predictive models was only 0.72. This was explained by the fact that the data used in the model establishment mostly included female subjects, and the average age was high. Khannara et al. [44] studied the effects of hypertension and diabetes on CKD progression by analysing common risk factors and using ANN, k-NN, and NB methods. Some studies tried to test urinary biomarkers such as urinary kidney injury molecule-1 (uKIM-1) and urinary neutrophil gelatinase-associated lipocalin (UNGAL) to predict the status of eGFR; however, they were not successful [45, 46]. Thus, researchers tried to use and combine easily available parameters for prediction, and they validated the model performance in both CKD to ESRD [4] or AKI to advanced CKD [8]. These models included the variables of older age, female sex, higher baseline serum creatinine value, and albuminuria, which are all available in the outpatient department. In addition to albumin, serum creatinine and EGFR, we also identified TG and LDL as prediction factors in our models. It was also previously reported that a distinct panel of lipid-related features may improve the prediction of CKD progression beyond EGFR and proteinuria [47].

Machine learning algorithms can build complex models and make accurate decisions when given relevant data. When there is an adequate amount of data, the performance of machine learning algorithms is expected to be sufficiently satisfactory. However, in specific applications, the data are often insufficient. Therefore, it is important to analyse these algorithms and obtain good results with a relatively small sample size. In this study, although we employed a relatively small dataset with 551 patients, the sample size satisfied the power analysis and identified that the linear models performed better than the other types of models.

It is expected that the existing sample set may not be able to support the solution because the training set is limited. In the case of low data dimensions, a linear classifier can separate samples more ideally, while more complex machine learning models such as SVM have more powerful learning but are also more prone to overfitting, resulting in a less accurate prediction. As shown above, k-NN performed the worst in our case. This is because k-NN is very sensitive to the number of data samples and neighbours. Therefore, the overall comparison shows that the linear models performed better in our study.

Finally, this study used non-urine indicators as clinical predictors and developed a web tool. The outpatients can be quickly screened to assist the physician in making decisions and provide patients with further proper examination and treatment. However, this study also has limitations. The sample size used is relatively small, and the parameters during tuning could be further optimized to avoid overfitting.

To further improve the accuracy of the established model, in subsequent research, more clinical data will be collected in our cohort, and the parameters will be further optimized. We are also establishing a Lasso-based predicted proteinuria range, which provides doctors and patients with more intuitive predictions. With the increase of users and data collected on our website, CKD research and patients can benefit in future clinical practices.

## Conclusions

In this study we established and compared nine models to predict the CKD severity using easily available clinical features during out-patient follow-up, finding that linear models including Elastic Net, Lasso, Ridge and LR showed the highest overall predictive power. We also identified that ALB, Scr, TG, LDL and EGFR had important impacts on the predictability of the models, while other predictors such as CRP, HDL and SNA were less important. The online tool developed can facilitate the prediction of proteinuria progress during follow-up practice.

## Additional file

**Additional file 1.** Model establishment and source codes brief illustrations.

## Abbreviations

LR: logistic regression
Ridge: ridge regression
Lasso: lasso regression
SVM: support vector machine
RF: random forests
k-NN: k-nearest neighbors
NN: neural networks
XGBoost: eXtreme Gradient Boosting
NB: naive Bayes
CKD: chronic kidney disease
RBF: Radial Basis Function
CRP: C-reactive protein
ALB: albumin
TC: total cholesterol
TG: triglyceride
BG: blood glucose
BUN: blood urea nitrogen
EGFR: estimated Glomerular Filtration Rate
Scr: serum creatinine
SUA: serum uric acid
SK: serum potassium
Sna: serum sodium
LDL: low-density lipoprotein
HDL: high-density lipoprotein
uprotein: urine protein
TP: true positive
FP: false positive
AU-ROC (AUC): area under the ROC curve
AP: average precision
ROC: receiver operating characteristic
PR: precision recall
CART: classification and regression tree
MSE: mean square error
PHP: hypertext preprocessor
BMI: body mass index
HTML5: HyperText Markup Language 5

## References

[1] Go AS, Chertow GM, Fan D, McCulloch CE (2004). Hsu C-y: Chronic kidney disease and the risks of death, cardiovascular events, and hospitalization. N Engl J Med.

[2] Levey AS, Tangri N, Stevens LA (2011). Classification of chronic kidney disease: a step forward. Ann Intern Med.

[3] Taal M, Brenner B (2008). Renal risk scores: progress and prospects. Kidney Int.

[4] Tangri N, Stevens LA, Griffith J, Tighiouart H, Djurdjev O, Naimark D, Levin A, Levey AS (2011). A predictive model for progression of chronic kidney disease to kidney failure. JAMA.

[5] Oliver MJ, Quinn RR, Garg AX, Kim SJ, Wald R, Paterson JM (2012). Likelihood of starting dialysis after incident fistula creation. Clin J Am Soc Nephrol..

[6] O’Hare AM, Choi AI, Bertenthal D, Bacchetti P, Garg AX, Kaufman JS, Walter LC, Mehta KM, Steinman MA, Allon M (2007). Age affects outcomes in chronic kidney disease. J Am Soc Nephrol.

[7] Wojciechowski P, Tangri N, Rigatto C, Komenda P (2016). Risk prediction in CKD: the rational alignment of health care resources in CKD 4/5 care. Adv Chronic Kidney Dis.

[8] Provenzano M, Chiodini P, Minutolo R, Zoccali C, Bellizzi V, Conte G, Locatelli F, Tripepi G, Del Vecchio L, Mallamaci F (2018). Reclassification of chronic kidney disease patients for end-stage renal disease risk by proteinuria indexed to estimated glomerular filtration rate: multicentre prospective study in nephrology clinics. Nephrol Dial Transpl..

[9] Everitt B, Hothorn T (2011). An introduction to applied multivariate analysis with R.

[10] Mendenhall WM, Sincich TL, Boudreau NS (2016). Statistics for engineering and the sciences, student solutions manual.

[11] Aho KA (2016). Foundational and applied statistics for biologists using R.

[12] Glantz SA, Slinker BK, Neilands TB (1990). Primer of applied regression and analysis of variance.

[13] Spiegel M, Stephens L (2014). Schaum’s outline of statistics.

[14] Menard S (2002). Applied logistic regression analysis.

[15] Meadows K, Gibbens R, Gerrard C, Vuylsteke A (2018). Prediction of patient length of stay on the intensive care unit following cardiac surgery: a logistic regression analysis based on the cardiac operative mortality risk calculator, EuroSCORE. J Cardiothorac Vasc Anesth..

[16] Kim S-J, Koh K, Lustig M, Boyd S, Gorinevsky D (2007). An interior-point method for large-scale $\ell_1 $-regularized least squares. IEEE J Select Top Signal Process.

[17] Friedman J, Hastie T, Tibshirani R (2010). Regularization paths for generalized linear models via coordinate descent. J Stat Softw.

[18] Marafino BJ, Boscardin WJ, Dudley RA (2015). Efficient and sparse feature selection for biomedical text classification via the elastic net: application to ICU risk stratification from nursing notes. J Biomed Inform.

[19] Tibshirani R (1996). Regression shrinkage and selection via the lasso. J R Stat Soc Ser B..

[20] Tikhonov AN, Goncharsky A, Stepanov V, Yagola AG (2013). Numerical methods for the solution of ill-posed problems.

[21] Hoerl AE, Kennard RW (1970). Ridge regression: biased estimation for nonorthogonal problems. Technometrics.

[22] Wan S, Mak M-W, Kung S-Y (2014). R3P-Loc: a compact multi-label predictor using ridge regression and random projection for protein subcellular localization. J Theor Biol.

[23] Nigrin A (1993). Neural networks for pattern recognition. Agri Eng Int Cigr J Sci Res Devel Manusc Pm.

[24] Salekin A, Stankovic J: Detection of chronic kidney disease and selecting important predictive attributes. In: IEEE Healthcare Informatics (ICHI), 2016 IEEE International Conference on. 2016. p. 262–70.

[25] Cortes C, Vapnik V (1995). Support-vector networks. Mach Learn.

[26] Dolatabadi AD, Khadem SEZ, Asl BM (2017). Automated diagnosis of coronary artery disease (CAD) patients using optimized SVM. Comput Methods Programs Biomed.

[27] Breiman L (2001). Random forests. Mach Learn.

[28] Ho TK. Random decision forests. In: Document analysis and recognition, 1995, proceedings of the third international conference on. IEEE; 1995. p. 278–282.

[29] Asaoka R, Hirasawa K, Iwase A, Fujino Y, Murata H, Shoji N, Araie M (2017). Validating the usefulness of the “random forests” classifier to diagnose early glaucoma with optical coherence tomography. Am J Ophthalmol.

[30] Chen T, Guestrin C: Xgboost: A scalable tree boosting system. In: Proceedings of the 22nd ACM sigkdd international conference on knowledge discovery and data mining. ACM; 2016. p. 785–94.

[31] Chen T, He T, Benesty M (2015). Xgboost: extreme gradient boosting. R package version.

[32] Zhang HX, Guo H, Wang JX (2018). Research on type 2 diabetes mellitus precise prediction models based on XGBoost algorithm. Chin J Lab Diagn..

[33] Bhuvaneswari P, Therese AB (2015). Detection of cancer in lung with k-nn classification using genetic algorithm. Procedia Mater Sci.

[34] Altman NS (1992). An introduction to kernel and nearest-neighbor nonparametric regression. Am Stat.

[35] Heaton J (2018). Ian goodfellow, yoshua bengio, and aaron courville: deep learning. Genet Program Evolvable Mach.

[36] Murphy KP (2012). Machine learning: a probabilistic perspective.

[37] Kuhn M, Johnson K (2013). Applied predictive modeling.

[38] Flach P, Kull M. Precision-recall-gain curves: Pr analysis done right. In: Advances in neural information processing systems. 2015. p. 838–46.

[39] Cerqueira DC, Soares CM, Silva VR, Magalhães JO, Barcelos IP, Duarte MG, Pinheiro SV, Colosimo EA, e Silva ACS, Oliveira EA (2014). A predictive model of progression of ckd to esrd in a predialysis pediatric interdisciplinary program. Clin J Am Soc Nephrol.

[40] Herget-Rosenthal S, Dehnen D, Kribben A, Quellmann T (2013). Progressive chronic kidney disease in primary care: modifiable risk factors and predictive model. Prev Med.

[41] Usui T, Kanda E, Iseki C, Iseki K, Kashihara N, Nangaku M (2017). Observation period for changes in proteinuria and risk prediction of end-stage renal disease in general population. Nephrology.

[42] Garlo KG, White WB, Bakris GL, Zannad F, Wilson CA, Kupfer S, Vaduganathan M, Morrow DA, Cannon CP, Charytan DM (2018). Kidney biomarkers and decline in eGFR in patients with type 2 diabetes. Clin J Am Soc Nephrol.

[43] Hsu CY, Xie D, Waikar SS, Bonventre JV, Zhang X, Sabbisetti V, Mifflin TE, Coresh J, Diamantidis CJ, He J, Lora CM (2017). Urine biomarkers of tubular injury do not improve on the clinical model predicting chronic kidney disease progression. Kidney Int.

[44] Afshinnia F, Rajendiran TM, Karnovsky A, Soni T, Wang X, Xie D, Yang W, Shafi T, Weir MR, He J (2016). Lipidomic signature of progression of chronic kidney disease in the chronic renal insufficiency cohort. Kidney Int Rep.

[45] Lin LJ, Chen XQ, Lin-Hong WU, Wei-Wei FU, Long ZP, Nephrology DO, Hospital P (2015). Blood pressure control on the progression of renal function in elderly patients with chronic kidney disease. China J Modern Med.

[46] Chase HS, Hirsch JS, Mohan S, Rao MK, Radhakrishnan J (2014). Presence of early CKD-related metabolic complications predict progression of stage 3 CKD: a case–controlled study. BMC Nephrol.

[47] Khannara Warangkana, Iam-On Natthakan, Boongoen Tossapon (2015). Predicting Duration of CKD Progression in Patients with Hypertension and Diabetes. Proceedings in Adaptation, Learning and Optimization.

