# Supplementary material for: Comparison and development of machine learning tools in the prediction of chronic kidney disease progression
Source: J Transl Med. 2019 Apr 11;17:119. doi: 10.1186/s12967-019-1860-0 (PMC6458616; doi:10.1186/s12967-019-1860-0)
Supplement: Supplementary file 1 — Additional file 1. Model establishment and source codes brief illustrations. [file 12967_2019_1860_MOESM1_ESM.docx]

**Additional file 1. Model establishment and source codes brief illustrations.**

**Performance metrics**

In the current study, the following metrics were applied to evaluate the performance of each model.

i.) TP (true positive), positive diagnoses classified as positive outcomes.

ii.) FP (false positive), negative diagnoses classified as positive outcomes.

iii.) TN (true negative), negative diagnoses classified as negative outcomes.

iv.) FN (false negative), positive diagnoses classified as negative outcomes.

v.) Confusion Matrix, was adopted to assess the classification performance. (Table S1)

vi.) Accuracy, to identify the diagnosis ability to correctly classify the dataset (Eq.1)

| $Accuracy=\frac{TP+TN}{TP+FP+TN+FN}$ | (1) |
| --- | --- |

vii.) TPR (true positive rate), or Sensitivity (Eq.2)

| $TPR=\mathrm{Re}call=Sensitivity=\frac{TP}{TP+FN}$ | (2) |
| --- | --- |

viii.) FPR (false positive rate), or type I error probability (Eq.3)

| $FPR=FP/(FP+TN)$ | (3) |
| --- | --- |

ix.) TNR (true negative rate), or Specificity (Eq.4)

| $Specificity=TNR=\frac{TN}{TN+FP}=1-FPR$ | (4) |
| --- | --- |

x.) Precision (Eq.5)

| $\Pr ecision=\frac{TP}{TP+FP}$ | (5) |
| --- | --- |

xi.) ROC (Receiver Operating Characteristic) curve, a curve determined by TPR and FPR for evaluating the model performance.

xii.) AUC (Area Under Curve), an index to evaluate the predictive and classification performance of a model.

xiii.) *F_1_* score, or balanced F Score, to measure the accuracy of a model. (Eq.6)

| $F_{1}=2\times\frac{\mathrm{Re}call\times\Pr ecision}{\mathrm{Re}call+\Pr ecision}$ | (6) |
| --- | --- |

xiv.) AP (Average precision), a weighted mean of precisions when achieved at a certain threshold. (Eq.7)

| $AP=\sum_{n} (R_{n}-R_{n-1})P_{n}$ | (7) |
| --- | --- |

Where *P_n_* (precision) and *R_n_* (recall) are at the nth threshold. (*R_k_*, *P_k_*) denotes an operating point.

xv.) Log-loss, or cross-entropy loss, to measure the performance of a classification. (Eq.8)

| $L(Y,P(Y\vert X))=-\log P(Y\vert X)=-\frac{1}{N}\sum_{i=1}^{N} (y_{i}\log p_{i}+(1-y_{i})\log(1-p_{i}))$ | (8) |
| --- | --- |

Where Y is output variable, X is input variable, L is loss function. N is the sample size, *P_i_* is the probability of *X_i_* are classified as 1.

**Model establishment and brief illustrations**

i.) Supporting vector machine, SVM: SVM is a machine learning approach proposed to solve high-dimension, small sample and non-linear datasets ^[1]^. (Eq.9)

| $f(X_{i})=\sum_{i=1}^{nSV} (a_{i}^{*}-a_{i})K(X_{i},X_{j}^{'})+b$ | （9） |
| --- | --- |

where $X_{i}$ stands for the$i$ independent variable, $f(X_{i})$ is outcome of dependent variable, $nSV$ is supporting vector, $a_{i}$ and $a_{i}^{*}$ are Lagrangian multipliers, $K(X_{i},X_{j}^{'})$is kernel function, and b is a constant.

ii.) Random forest, RF^[2]^: RF is a type of classification and regression tree (CART)[3], established by a series of decision making models $\{h(X,\theta_{k},k=1,2,...)\}$, where random variable $\{\theta_{k}\}$ is independent identically distributed. (Eq.10)

| $H(x)=\max_{Y} \sum_{i=1}^{K} I(h_{i}(x))=Y$ | （10） |
| --- | --- |

Where $H(x)$ is RF classification result, $Y$ is target variable, $h_{i}(x)$ denotes the results of $i$_th_ decision tree, *I(h_i_(x))* is an indicator function.

iii.) Ridge regression, RR: RR is a multiple linear regression which integrates the two norm regularization into the least squares model [4]. (Eq.11)

| $(\alpha,\beta)=\mathrm{argmin}(\sum_{i=1}^{n} (y_{i}-\alpha_{i}-X_{i}\beta)^{2}+\lambda\vert\vert\beta\vert\vert_{2}^{2})$ | （11） |
| --- | --- |

Where $X_{i}$ is the $i$_th_ independent variable, $\alpha$and $\beta$ are regression coefficient, $||\beta||_{2}$ denotes two norm (), $y_{i}$ is the outcome value of $X_{i}$, $n$ is the number of sample used in the model, $\lambda$is the penalty parameter.

iv.) Lasso: Lasso is similar to RR which was proposed to the interpretability of subset selection and the stability of ridge regression[5]. Lasso adds a one norm constraint on the regression coefficient and adds penalty to the absolute form. (Eq.12)

| $(\alpha,\beta)=\mathrm{argmin}(\sum_{i=1}^{n} (y_{i}-\alpha_{i}-X_{i}\beta)^{2}+\chi\vert\vert\beta\vert\vert_{1})$ | （12） |
| --- | --- |

Where $X_{i}$ is the $i$_th_ independent variable, $\alpha$and $\beta$ are regression coefficient, $||\beta||_{1}$ denotes one norm (), $y_{i}$ is the outcome value of $X_{i}$, $n$ is the number of sample used in the model, $\lambda$is the penalty parameter.

v.) Elastic Net, EN: Elastic Net is a regression method that linearly combines the and penalties of the lasso and ridge methods[6, 7]. (Eq.13)

| $(\alpha,\beta)=\mathrm{argmin}(\sum_{i=1}^{n} (y_{i}-\alpha_{i}-X_{i}\beta)^{2}+\chi\vert\vert\beta\vert\vert_{1}+\lambda\vert\vert\beta\vert\vert_{2}^{2})$ | （13） |
| --- | --- |

Where $X_{i}$ is the $i$_th_ independent variable, $\alpha$and $\beta$ are regression coefficient, $||\beta||_{1}$ denotes one norm (), $||\beta||_{2}$ denotes two norm (), $y_{i}$ is the outcome value of $X_{i}$, $n$ is the number of sample used in the model, $\lambda$is the penalty parameter.

vi.)k-nearest neighbors, k-NN: is a non-parametric method for classification. (Eq.14)

| $\hat{y}(x)=\frac{1}{k}\sum_{x_{i}\in N_{k}(x)} y_{i}$ | （14） |
| --- | --- |

Where $N_{k}(x)$ is training data of k nearest points to variable x.

vii.) XGBoost: This gradient boosting classifier is a recently proposed model [8, 9], which utilized second-order Taylor expansion of the loss function.

viii.) Logistic regression, LR: This is a general regression model [10]：(Eq.15)

| $Logit(P)=\alpha+\beta_{i}X_{i}$ | （15） |
| --- | --- |

Where $\alpha$is a constant, $\beta_{i}$ denotes regression coefficient, $X_{i}$ is the $i$_th_ independent variable, P stands for probability.

ix.) Neural network, NN: Different from linear and logistic regression, there are hidden layers between input and output lay [11]. In order to avoid over-fitting problems, the neural network adds two norm regularization () coefficients to participate in model training.

**Additional files and source codes**

#All attached source codes and result files were packed in “SupplementaryFiles.zip” for reference.

/datasets/uProt.csv

#This file contains raw clinical dataset.

/code

#This folder contains all modeling codes based on Python and R language. After running codes, the results will be saved in the “/result” folder including AUC values, importance, prediction values and true values. The three “.py” files named with “_model” are used to build online predictive tools for HTML display.

/result

#“*threshold” files contain threshold of sampling in each model.

#“*data” files contain predicted output, true value, AUC and importance of each feature. In sheet “data”, the column stand for features while rows stand for predicted values during sampling. In sheet “y_testdata”, the column stands for true values in one sampling. In sheet “y_prodata”, the column stands for predicted values in the corresponding “y_testdata” sheet.

#“allmodel_auc1030.csv” file contains XXXXX

#“heatmap.xlsx” contains predicted values of nine models and true values.

/web

#This folder contains source codes for Web based predictive tools.

/web/view

#This folder contains front end interfaces.

/web/model

#This folder contains PHP code for system backstage.

/web/Pythonmodel_and_Callpython

This folder contains modeling files and “popen” based Python-PHP communication files.

**References**

1. Burges CJ: **A tutorial on support vector machines for pattern recognition.** *Data mining and knowledge discovery* 1998, **2:**121-167.

2. Breiman L: **Random Forests.** *Machine Learning* 2001, **45:**5-32.

3. Breiman L: *Classification and regression trees.* Routledge; 2017.

4. Hoerl AE, Kennard RW: **Ridge regression: Biased estimation for nonorthogonal problems.** *Technometrics* 1970, **12:**55-67.

5. Tibshirani R: **Regression shrinkage and selection via the lasso.** *Journal of the Royal Statistical Society Series B (Methodological)* 1996**:**267-288.

6. Friedman J, Hastie T, Tibshirani R: **Regularization paths for generalized linear models via coordinate descent.** *Journal of statistical software* 2010, **33:**1-22.

7. Kim S-J, Koh K, Lustig M, Boyd S, Gorinevsky D: **An Interior-Point Method for Large-Scale $\ell_1 $-Regularized Least Squares.** *IEEE journal of selected topics in signal processing* 2007, **1:**606-617.

8. Chen T, He T, Benesty M: **Xgboost: extreme gradient boosting.** *R package version 04-2* 2015**:**1-4.

9. Chen T, Guestrin C: **Xgboost: A scalable tree boosting system.** In *Proceedings of the 22nd acm sigkdd international conference on knowledge discovery and data mining*. ACM; 2016: 785-794.

10. Hosmer Jr DW, Lemeshow S, Sturdivant RX: *Applied logistic regression.* John Wiley & Sons; 2013.

11. Bishop CM: *Neural networks for pattern recognition.* Oxford university press; 1995.

Additional figure


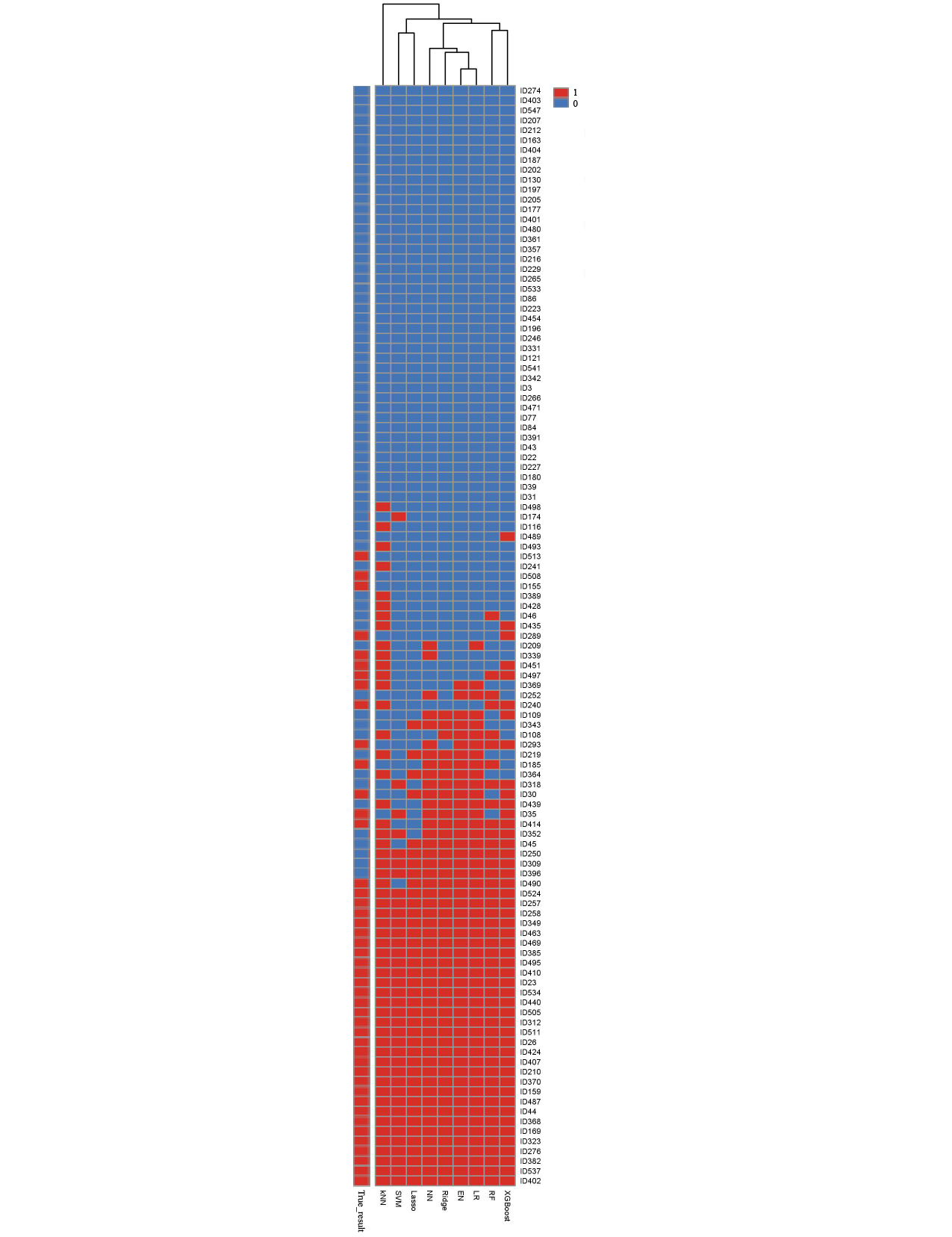


Figure S1. Hierarchical clustering analysis was carried out based on nine methods using false positive (FP) and false negative (FN) from 110 validation dataset. Models with similar method and principle are closely clustered.

Additional tables

Table S1. Confusion matrix

| Confusion matrix | | Prediction | |
| --- | --- | --- | --- |
|  |  | Negative | Positive |
| Actural | Negative | TN | FP |
|  | Positive | FN | TP |

Table S2. File names and their functions in “/code” folder

| File name | Description |
| --- | --- |
| use_Threshold.py | Confusion matrices of 9 models -Table 3 |
| pROC_auc.R | Performance summary-Table 4 |
| pROC_compare.R | Comparison of AUCs-Table 5 |
| AUC-mean.py | Mean ROC curves-Figure 3 (a) |
| Precision_Recall_mean.py | Mean PR curves -Figure 3 (b) |
| boxplot.py | Box plots-Figure 3 (c) |
| heatmap.R | Hierarchical clustering-Figure S1 |
| Model_Importance_Chart.py | Importance of models –Figure 4 |
| pROC_thresholds.R | AUC cutoff calculation |
